# Rationale for targeting complement in COVID‐19

**DOI:** 10.15252/emmm.202012642

**Published:** 2020-07-12

**Authors:** Anastasia Polycarpou, Mark Howard, Conrad A Farrar, Roseanna Greenlaw, Giorgia Fanelli, Russell Wallis, Linda S Klavinskis, Steven Sacks

**Affiliations:** ^1^ MRC Centre of Transplantation Peter Gorer Department of Immunobiology School of Immunology and Microbial Sciences Guy's Hospital King's College London London UK; ^2^ Department of Respiratory Science and Infection Leicester Institute of Chemical and Structural Biology University of Leicester Leicester UK; ^3^ Department of Infectious Diseases School of Immunology and Microbial Sciences Guy's Hospital King's College London London UK

**Keywords:** complement proteins, COVID‐19, lectin pathway, SARS‐CoV‐2, therapeutics, Immunology, Microbiology, Virology & Host Pathogen Interaction

## Abstract

A novel coronavirus, SARS‐CoV‐2, has recently emerged in China and spread internationally, posing a health emergency to the global community. COVID‐19 caused by SARS‐CoV‐2 is associated with an acute respiratory illness that varies from mild to the life‐threatening acute respiratory distress syndrome (ARDS). The complement system is part of the innate immune arsenal against pathogens, in which many viruses can evade or employ to mediate cell entry. The immunopathology and acute lung injury orchestrated through the influx of pro‐inflammatory macrophages and neutrophils can be directly activated by complement components to prime an overzealous cytokine storm. The manifestations of severe COVID‐19 such as the ARDS, sepsis and multiorgan failure have an established relationship with activation of the complement cascade. We have collected evidence from all the current studies we are aware of on SARS‐CoV‐2 immunopathogenesis and the preceding literature on SARS‐CoV‐1 and MERS‐CoV infection linking severe COVID‐19 disease directly with dysfunction of the complement pathways. This information lends support for a therapeutic anti‐inflammatory strategy against complement, where a number of clinically ready potential therapeutic agents are available.

## Graphical abstract

This review offers a balanced view on how the complement system may be engaged in COVID‐19 and suggests therapeutic strategies, some of which already in clinical trials.

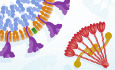

**Glossary**
Acute respiratory distress syndrome (ARDS): The acute respiratory distress syndrome (ARDS) is a clinical syndrome defined by acute onset hypoxaemia and bilateral pulmonary opacities not fully explained by cardiac failure or volume overload. The syndrome can be triggered by pulmonary or extrapulmonary sepsis, aspiration, trauma, blood product transfusion or pancreatitis.
Antibody‐dependent enhancement (ADE): A process where virus entry is facilitated by the interaction of virus‐specific antibody complexes to Fc and/or complement receptors expressed on the surface of immune cells including macrophages, neutrophils, mast cells, natural killer cells and B cells.
C1q: A part of the C1 protein complex that binds to antigen–antibody complexes and initiates the classical pathway of complement activation.
C3: The common component of the three complement activation pathways, cleaved by C3‐converting enzyme complexes (C3 convertases) into its active fragments C3a and C3b.
C5: A component of the terminal pathway of complement activation, cleaved by C5 convertases into C5a and C5b.
C5b‐9 (membrane attack complex): Innate immune effector of the terminal complement pathway formed by interaction of C5b, C6, C7, C8 and C9. It forms cytotoxic pores on the surface of pathogens.
Chemokine: A family of small chemoattractant cytokines that can induce direct chemotaxis.
Coagulation pathway: A cascade of enzyme activation events that mediate polymerisation of fibrin and activation of platelets leading to blood clot formation. It contains two different pathways by which the blood‐clotting cascade is initiated in haemostasis and pathological thrombosis, i.e. the tissue factor pathway (extrinsic) and the contact pathway (intrinsic).
Collectins: Collagen‐containing C‐type lectins that have globular carbohydrate recognition domains (CRDs) that display binding affinity to a variety of glycan ligands. Collectins can act as PRR for the lectin pathway of complement activation and form complexes with MASPs. Nine collectins have been discovered to date.
Complement anaphylatoxins (C3a, C4a, C5a): Complement system small polypeptides produced after proteolytic cleavage of large glycoproteins C3, C4 and C5 by convertases in response to complement activation. They have pleiotropic biological effects such as involvement in inflammation, cell apoptosis, tissue regeneration and fibrosis, lipid metabolism, vasodilation, innate and adaptive immune responses through acting via specific receptors on the surface of immune and non‐immune cells.
Complement system: A network of at least 40 proteins that is part of the innate immune system and complements the ability of antibody and phagocytes to eliminate pathogens though marking pathogens for phagocytic clearance, mediating pathogen lysis though pore formation and recruiting inflammatory cells to the site of infection. It also enhances and directs the adaptive arm immune response comprised of T cells and B cells. Complement activation includes the classical, the lectin and the alternative pathway. All three pathways converge at the complement component C3 and lead to C3b deposition on the surface of an invading pathogen.
COVID‐19: The infectious disease caused by SARS‐CoV‐2.
Cytokine: Cell signalling protein molecules with a wide range of biological functions.
Factor VIII: An essential blood‐clotting protein which participates in coagulation. Factor VIII is deficient or defective in patients with classical haemophilia and von Willebrand syndrome.
Ficolins: PRRs for the lectin pathway containing a collagen‐like domain and a fibrinogen‐like domain that has a specific binding affinity for N‐acetylglucosamine. They can act as opsonins and complex with MASPs to activate the complement pathway. Three types of ficolins are known to date: M‐ficolin (ficolin‐1), L‐ficolin (ficolin‐2) and H‐ficolin (ficolin‐3).
Glycan: Polysaccharides or carbohydrate‐based polymers.
Hyper‐cytokinaemia/cytokine storm: An excessive innate immune response characterised by overproduction of cytokines and chemokines into the blood in a short time.
Lectin pathway: A complement pathway which has multiple pattern recognition receptors (PRRs) such as collectins and ficolins.
MBL‐associated serine proteases (MASPs): The associated serine proteases of the lectin system which form complexes with the PRRs such as collectins and ficolins activating the lectin pathway.
MERS‐CoV: A pathogenic coronavirus causing the Middle East respiratory syndrome (MERS), also known as camel flu which first occurred in 2012 in Saudi Arabia.
Neutrophilia: Increase in the absolute neutrophil count in peripheral blood above 7.5 × 10^9^/L. This can be due to a reaction to infection, inflammation, stress, medication or malignancy or due to primary abnormalities from bone marrow.
Pattern recognition molecule (PRM): Proteins that recognise molecules found in pathogens (pathogen‐associated molecular patterns or PAMPs) or molecules released by damaged cells (damage‐associated molecular patterns or DAMPs). PRR can be soluble or cell‐bound.
SARS‐CoV‐2: The coronavirus which causes severe acute respiratory syndrome in COVID‐19, which emerged in December 2019 in Wuhan, China, causing a global pandemic. Previously known as 2019‐nCoV.
Thrombin‐activatable fibrinolysis inhibitor (TAFI): A circulating enzyme that protects the clot against fibrinolysis. Increased activation or defect may lead to thrombin generation or bleeding. TAFI may also have an important role in the regulation of inflammation, wound healing and blood pressure.

## Introduction

Complement has evolved as a major defence against infection, evident by the fact that many microorganisms including bacteria and viruses have developed resistance to complement and can exploit the complement system to facilitate tissue invasion (reviewed in Agrawal *et al*, 2017). The inflammatory response to infection mediated by complement may also be tissue‐destructive and contribute to the clinical syndrome of sepsis (reviewed by Rittirsch *et al*, 2008) and multiorgan failure (MOF) (reviewed by Rittirsch *et al*, 2012). A tipping point occurs where the harmful effects of complement in the response to infection may outweigh the beneficial effects. This is highly pertinent in COVID‐19 infection, in which the highest mortality is evident in patients with severe pneumonitis, systemic sepsis and MOF (Li *et al*, 2020; Poston *et al*, 2020) and where evidence of profound complement activation is beginning to emerge. The aim of this review is to summarise current understanding of the interaction of SARS‐CoV‐2 virus with the complement system and examine the case for targeting the inflammatory reaction mediated by complement in severe COVID‐19 disease. Since complement is a compartmentalised component of innate immunity, this review will take into account the local synthesis of complement components at the portal of viral invasion, as well as the circulating pool. Necessarily, the review will focus on the recent literature, albeit fragmentary, and draw inference from the larger body of published work on other coronavirus pathogens.

## The SARS‐CoV‐2 virus

SARS‐CoV‐2 is a human coronavirus, first discovered in China in association with cases of severe acute respiratory syndrome (SARS) in late 2019 (Zhu *et al*, 2020). The virus is a member of the Coronaviridae, a diverse family of enveloped positive‐strand RNA viruses that are defined by comparative sequence homology (Coronaviridae Study Group of the International Committee on Taxonomy of, 2020). The viruses are named after the appearance of glycoprotein spikes projecting from the virus surface that resemble a solar corona by electron microscopy (Bingham & Almeida, 1977). Of the Coronaviridae, only members of the so‐called Alphacoronavirus and Betacoronavirus genera normally infect mammals (Cui *et al*, 2019). The majority of human coronaviruses (HoCoV) that belong to these genera including HoCoV‐229E, HoCoV‐NL63, HoCoV‐OC43 and HKU1, which induce mild seasonal respiratory disease, referred to as the “common cold” in immunocompetent individuals (Dijkman *et al*, 2012; Corman *et al*, 2019). However, since 2002, highly pathogenic coronaviruses for humans have emerged, including SARS‐CoV‐1 (Peiris *et al*, 2003b), Middle East respiratory syndrome‐CoV (MERS‐CoV) (Zaki *et al*, 2012) and SARS‐CoV‐2 (Zhu *et al*, 2020) that pose a significant public health risk. On the basis of sequence analysis, these pathogenic coronaviruses have animal origins, with bats implicated as the natural reservoir of SARS‐CoV‐1 and SARS‐CoV‐2 with other animals serving as potential intermediate hosts (Li *et al*, 2005; Andersen *et al*, 2020). Of note, humans are opportunistic hosts for these viruses, a fact that may underlie the pathology reported in human infection.

**Figure 1:**
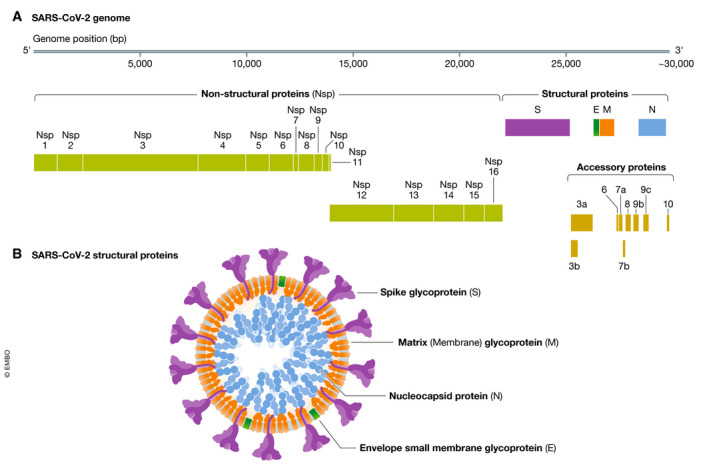
**Genome and proteins of SARS‐CoV‐2**.

**Figure 2:**
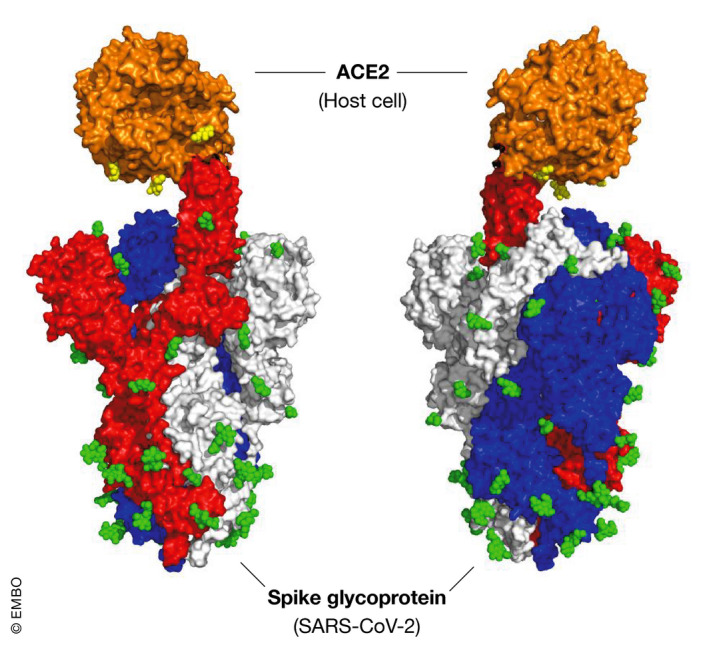
Model of SARS‐CoV‐2 spike protein and glycosylation sites The three protomers of the spike are shown in red, blue and white. The host cell receptor ACE‐2 is in orange. N‐glycosylation sites are displayed in green (on the spike) or yellow (on the receptor). Each cluster of spheres represents a single N‐acetylglucosamine residue (one sphere per atom), though the actual N‐linked glycan will consist of multiple sugar residues at each of the glycosylation sites. It can be seen that the spike has multiple N‐linked glycosylation sites (while the receptor only has three N‐linked sites). The model was generated by superposing structures PDB:6M0J (Lan *et al*, 2020) and PDB: 6VSB (Wrapp *et al*, 2020).

Sequence analysis of the SARS‐CoV‐2 genome suggests it encodes at least 14 open reading frames (orf), with two‐thirds of the orfs at the 5′ end of the genome encoding non‐structural proteins associated with the replicase/transcriptase complex. The remaining orfs at the 3′ end encode for nine putative accessory proteins and four essential structural proteins: the spike (S), membrane (M) and envelope (E), that comprise the surface proteins and a nucleocapsid (N) protein bound to the RNA genome (Gordon *et al*, 2020; Zhou *et al*, 2020) (Fig 1). The S protein mediates attachment to host cell surface receptors and facilitates viral entry into the cytosol following proteolytic cleavage by a host membrane serine protease TMPRSS2, with fusion to endosomal membranes (Hoffmann *et al*, 2020). The S protein forms characteristic homotrimers protruding from the viral surface (Walls *et al*, 2020) and is enriched with a plethora of glycan signatures (preprint: Shajahan *et al*, 2020; preprint: Watanabe *et al*, 2020) (Fig 2). The SARS‐CoV‐2 S gene encodes 22N‐linked glycan sequons per protomer leading to an array of host‐derived glycans with each trimer displaying 66N‐linked glycosylation sites (preprint: Watanabe *et al*, 2020). By analogy with SARS‐CoV‐1, the M protein has a critical function in the trafficking and assembly of proteins incorporated into the virion (Ye & Hogue, 2007; Siu *et al*, 2008), being necessary for binding and packaging of the ribonucleoprotein complex and interaction with the structural proteins in virus budding (McBride *et al*, 2014). The N protein also plays a wider role in deregulating host cell function, via antagonism of interferon β production (Kopecky‐Bromberg *et al*, 2007), modulation of the cell cycle regulation (Surjit *et al*, 2005) and host translational shutoff (Zhou *et al*, 2008) that taken together may contribute to disease pathogenesis.

## The immune pathogenesis of SARS‐CoV‐2

SARS‐CoV‐2, like SARS‐CoV‐1, utilises angiotensin‐converting enzyme 2 (ACE2) as an entry receptor (Hoffmann *et al*, 2020), which suggests SARS‐CoV‐2 shares a similar tropism for alveolar type II epithelial cells and possibly resident alveolar macrophages that express this receptor. The binding of the S glycoprotein to ACE2 downregulates the receptor and increases production of angiotensin II, which stimulates type 1A angiotensin receptor (AGTR1A) (Imai *et al*, 2005). This increases pulmonary vascular permeability and lung pathology (Imai *et al*, 2005). ACE2 is also expressed by a minimal percentage of peripheral blood monocytes (Jiang *et al*, 2005). However, whether SARS‐CoV‐2 directly infects any innate immune leucocytes remains unknown.

It is highly possible that SARS‐CoV‐2 may co‐opt other entry receptors or may employ other modes of cellular entry, such as antibody‐dependent enhancement (ADE). For example, ADE may occur through the binding of virus–antibody immune complexes on Fc receptors or complement receptors or alternatively, by inducing a conformational change in envelope glycoproteins required for virus–cell membrane fusion (reviewed by (Tirado & Yoon, 2003)). Moreover, SARS‐CoV‐2, like SARS‐CoV‐1, may interact with the dendritic cell‐specific intercellular adhesion molecule‐grabbing nonintegrin (DC‐SIGN), a C‐type lectin present on myeloid dendritic cells (DCs), and the related protein DC‐SIGNR (also termed L‐SIGN) (Jeffers *et al*, 2004; Marzi *et al*, 2004; Yang *et al*, 2004). It is well established that these cell surface receptors engage carbohydrate ligands expressed by several viruses and by that mechanism enhance host cell entry and infection (Mitchell *et al*, 2001; Grove & Marsh, 2011). DCs are reported to transfer the SARS‐CoV‐1 to other susceptible target cells through a synapse‐like structure (Yang *et al*, 2004). Through this direct mechanism, DCs may act as a viral reservoir that may contribute to the chronicity of this infection (Yang *et al*, 2004). A recent study from Wang *et al* has shown that SARS‐CoV‐2 is able to directly infect T cells through the S glycoprotein (Wang *et al*, 2020b). Other receptors expressed on the surface of T cells, such as CD147, could mediate viral entry (Chen *et al*, 2020). Lymphopenia in COVID‐19 patients could also be explained by the high levels of the programmed cell death protein 1 (PD‐1) on CD8^+^ T cells (Moon, 2020), which is known to trigger T‐cell exhaustion (Jiang *et al*, 2015).

The finding that severe cases of COVID‐19 are less common in young children (Dong *et al*, 2020), while this age group exhibits highly effective innate immune responses (Nikolich‐Zugich, 2018), strongly suggests the crucial role of innate immunity in this disease. However, although the innate immune system can play an important protective role against invading pathogens (Takeuchi & Akira, 2009), when this response is overexpressed, it can contribute to immune‐mediated pathology in virus infections (Thiel & Weber, 2008; Henderson *et al*, 2020). For example, disease severity has been reported to increase during SARS‐CoV‐1 infection in the context of decreasing viral load (Peiris *et al*, 2003a). By analogy to SARS‐CoV‐1 (Gu *et al*, 2005), infection by SARS‐CoV‐2 is also characterised by neutrophilia, lymphopenia and hyper‐cytokinaemia (Mendez *et al*, 2019; Bermejo‐Martin *et al*, 2020). This “cytokine storm” induced by viral infection can then elicit inflammatory‐induced lung injury (Huang *et al*, 2020). A group of cytokines and chemokines have been associated in the literature with different coronaviral infections (IL‐5, IL‐6, IL‐12, IFN‐γ, G‐CSF, CXCL1, MCP1, TNF‐α), or specifically to SARS‐CoV‐2 (IL‐1β, IL‐2, IL‐6, IL‐8, IL‐10, IL‐17, IP10, MCP1, TNF‐α) (Huang *et al*, 2020; Qin *et al*, 2020; Xu *et al*, 2020). It is believed that the pathogenetic mechanism might involve a delayed type I interferon (IFN) production, resulting in the loss of viral control in the early phase of infection and influx of inflammatory immune cells, including monocytes/macrophages that hyper‐produce pro‐inflammatory cytokines in a similar way to SARS‐CoV‐1 (Yoshikawa *et al*, 2009). Added to this, comorbidities such as hypertension, diabetes, obesity, cardiovascular and respiratory diseases have all been associated with COVID‐19 severity and lethality (Yang *et al*, 2020). Pre‐existing inflammation and hypoxia associated with certain conditions can predispose the respiratory tract to viral infections (Amin *et al*, 2013; Kapur *et al*, 2014; Furuta *et al*, 2018).

## Ligand recognition by the complement system

The complement system is a major part of innate immunity and comprises a cascade of proteins that directly or indirectly destroy invading organisms and damaged cells, and interacts with the adaptive immune system (Turnberg & Botto, 2003). Activation of the complement system causes C3b—the large split fragment of the central component C3—to deposit on the activating surface. C3b‐opsonised cells can be removed by the phagocytic system or C3b may lead to the cleavage of C5 and to the formation of the membrane attack complex (MAC) C5b‐9, which results in cell injury and cell death. In addition, the small biologically active fragments C3a and C5a are anaphylatoxins, which recruit and activate leucocytes to promote inflammation.

**Figure 3:**
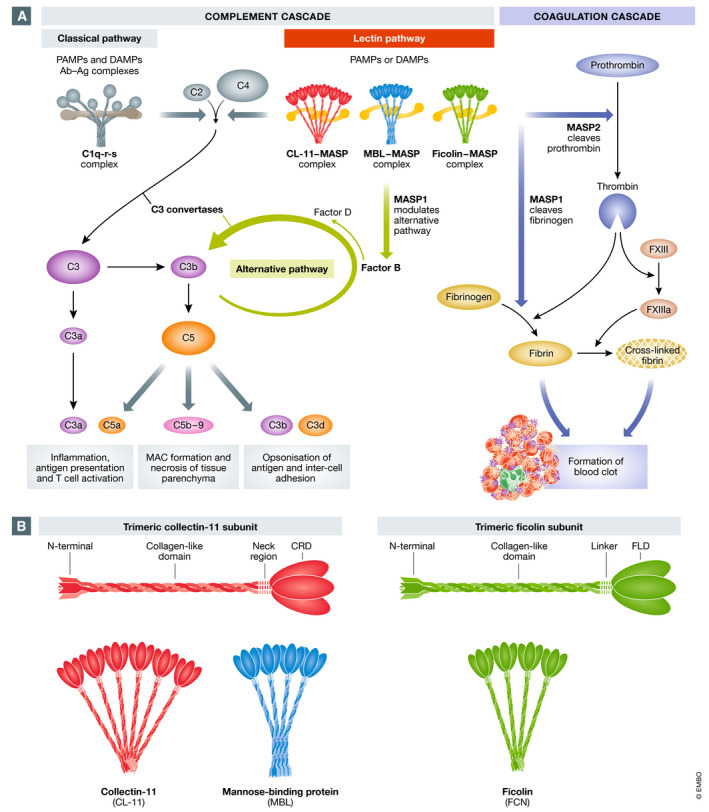
Models of the complement system (A) Simplified scheme of the complement and coagulation cascades and some of their interactions. The complement system comprises three main pathways: classical, lectin and alternative. The classical and lectin pathways are initiated through the action of pattern recognition molecules (PRM): C1q for the classical pathway; and collectins (e.g. CL‐11, collectin‐11; and MBL, mannose‐binding lectin) and ficolins for the lectin pathway. PRMs bind to pathogen‐associated molecular patterns (PAMPs) and damage‐associated molecular patterns (DAMPs). Following this, cleavage of complement factors C4 and C2 generates C3 convertase (C4bC2b), which cleaves C3 to C3a and C3b. C3b binds factor B, which is cleaved by factor D to generate C3bBb, the alternative pathway convertase, which results in amplification of C3b from C3. The two C5 convertases (C4bC2bC3b and C3bBbC3b) cleave C5 into C5a and C5b, the latter alongside C6, C7, C8 and C9 forming the membrane attack complex (MAC) C5b‐9. Meanwhile, the other products of C3 cleavage (C3a and the end metabolite of C3b called C3d) and C5 cleavage (C5a) have a number of roles including opsonisation, inflammation and the recruitment of the adaptive immune system. In the coagulation cascade, prothrombin is converted to thrombin, which in turn converts fibrinogen to fibrin and factor XIII to factor XIIIa. Fibrin forms the structure of the blood clot, while the factor XIIIa stabilises this clot by cross‐linking fibrin. Cross talk between the complement system and the coagulation system occurs through the actions of the MBL‐associated serine proteases (MASPs). MASP‐2 can convert prothrombin to thrombin, while MASP‐1 can act like thrombin and convert fibrinogen to fibrin (adapted from Nauser *et al*, 2018; Shimogawa *et al*, 2017). For a more extensive review of complement–coagulation interactions, see (Lupu *et al*, 2014). (B) Schematic representation of relevant lectin pathway pattern recognition molecules and their oligomeric structures. CL‐11 and ficolins initially form trimeric subunits, which then combine to form oligomers. MBL forms trimers and tetramers of MBL subunits, but both higher (pentamers and hexamers) and lower forms (monomers and dimers) also occur (adapted from Garred *et al*, 2016; Selman & Hansen, 2012). CRD: carbohydrate recognition domain; FLD: fibrinogen‐like domain.

Complement activation occurs by three main routes, the classical, lectin and alternative pathways, all of which converge on C3 (Fig 3A). The classical pathway uses the pattern recognition molecule (PRM) C1q that detects bound antibody or other immune surveillance molecules, such as C‐reactive protein. The lectin pathway uses a diverse set of PRMs including collectins and ficolins, which recognise carbohydrate structures on pathogens or injured host cells (reviewed in Howard *et al*, 2018). In contrast, the alternative pathway lacks a specific PRM but it can greatly amplify the amount of C3b formed by the classical or lectin pathways (reviewed by (Lachmann, 2018)). Direct hydrolysis of C3 may also trigger the alternative pathway.

The lectin pathway of complement is the most recent complement activation pathway to be described and is of particular interest in the setting of viral infection (Matsushita & Fujita, 1992). The pathway starts with ligand recognition by lectins known as collectins (e.g. mannose‐binding lectin [MBL]; collectin‐10 [CL‐10]; and collectin‐11 [CL‐11]) and ficolins (FCN 1, 2 and 3). The basic structures of these soluble collectins include a globular trimeric carbohydrate recognition domain (CRD) and a collagen‐like tail with a binding site for MBL‐associated serine proteases (MASPs 1, 2 and 3) (Fig 3B). Ficolin structures, on the other hand, feature a trimeric fibrinogen‐like CRD and a collagen‐like tail, which also binds to MASPs 1, 2 and 3 (Fig 3B). The lectin subunits form oligomers with increased avidity for ligand binding. These mammalian lectins differ in their preferred carbohydrate ligand, MBL, for example, binding with higher avidity to GlcNAc and D‐mannose, while CL‐11 has a higher avidity for L‐fucose and D‐mannose (Weis *et al*, 1992). Recognition of the preferred carbohydrate causes the lectin–MASP complex to initiate complement activation by cleavage of C3. Of the three essential MASPs, only MASP‐2 has been shown to directly cleave C3. The lectin–MASP complex can also directly stimulate alternative pathway activation (Iwaki *et al*, 2011). These lectins are involved in a range of immune functions including viral neutralisation and clearance and promotion of inflammation through complement‐dependent mechanisms and also by independent mechanisms that include calreticulin receptor binding (Kishore *et al*, 2006; Nayak *et al*, 2012).

A distinction can be made between the role of locally synthesised complement and that of the circulating pool secreted mainly by hepatocytes (Sacks & Zhou, 2012). Studies on C3 and CL‐11 produced within the kidney mainly by tubule epithelial cells have shown marked contribution to renal inflammatory injury, whereas the contribution of systemic components was negligible (Pratt *et al*, 2002; Farrar *et al*, 2006, 2016). In fact, many organs and cell types, including lung alveolar and bronchial epithelial cells as well as infiltrating leucocytes, produce a range of complement components despite these tissues being bathed in an abundant circulating pool (reviewed in Nauser *et al*, 2018). The reason for this local synthesis is unclear, although it could be because the large molecular size of proteins such as C3 (180 kDa) and CL‐11 (100‐200 kDa) limits their passage from the vascular compartment to extravascular sites at the portal of pathogen entry. The activity of complement in any tissue therefore is likely to involve a complex interplay between that produced by the tissue‐resident and migratory cells and components distributed from the plasma.

Lung tissue expresses several complement factors. CL‐11 and CL‐10 are detected by mRNA and protein analysis (Keshi *et al*, 2006; Motomura *et al*, 2008; Hansen *et al*, 2018). MBL is not detected in the lung but is an abundant component of the liver and circulation (Hansen *et al*, 2018). Type II alveolar cells also express FCN‐1 (Liu *et al*, 2005) and secrete the core complement components C3 and C5 as well as the classical/lectin pathway constituents C4 and C2 (Strunk *et al*, 1988; Pandya & Wilkes, 2014). Bronchial epithelium is another source of the pivotal component C3 (Varsano *et al*, 2000). It may be helpful to regard MBL as a guardian of the circulation, whereas other lectins like CL‐11 and FCN‐1 are located at epithelial surfaces too.

Within the vascular compartment, complement activation can promote endothelial injury and thrombosis. While this has largely been attributed to classical pathway (antibody‐mediated) activation, a role for the lectin pathway of complement activation has recently come to attention. MASP‐2 has been shown to cleave prothrombin to generate thrombin (Krarup *et al*, 2007), a serine protease that leads to the conversion of fibrinogen to fibrin—essential for clot formation. MASP‐1 behaves like thrombin in that it cleaves factor VIII and fibrinogen as well as thrombin‐activatable fibrinolysis inhibitor (TAFI) (Howard *et al*, 2018). Furthermore, both MBL‐MASP and ficolin–MASP complexes bound to glycan ligand can generate a blood clot similar to those generated by thrombin when provided with factor VIII and fibrinogen (Gulla *et al*, 2010). In this way, ligand recognition by the lectin complement pathway can signal to the coagulation pathway, linking these two arms of innate immunity (Fig 3A). The finding of elevated levels of CL‐11 in hypercoagulable states could underpin this ability to trigger coagulation in severely ill patients (Takahashi *et al*, 2014), as we shall now discuss.

## Complement involvement in acute respiratory distress syndrome

Complement activation is a common if not fixed feature of ARDS associated with infectious and non‐infectious causes. Characteristically, C5a is elevated in peripheral blood samples and has been proposed as a marker of ARDS associated with severe sepsis, cytokine storm and multiorgan failure (MOF) (Hammerschmidt *et al*, 1980). Polymorphonuclear neutrophil (PMN) aggregation within the injured lung predisposes towards the development of ARDS, coinciding with increase in the levels of C5a (Hammerschmidt *et al*, 1980). Furthermore, PMN exposed to activated C5a can adhere to and damage the vascular endothelium, leading to increased vascular permeability and the genesis of ARDS (Hammerschmidt *et al*, 1980). This is an important observation as it may explain the neutrophilia that has also been described during COVID‐19 (preprint: Zhang *et al*, 2020).

The complement system also interacts strongly with lung macrophages modifying their response to different pathogens (reviewed by Bohlson *et al*, 2014). Macrophages carry out complement effector functions by expressing numerous receptors that detect complement components, including CR1, CR3, CR4, C3aR and C5aR1 (Bohlson *et al*, 2014). Alveolar macrophages can also synthesise complement proteins, and lung macrophages have known ability to cleave C5 to generate C5a and downstream inflammatory signalling through C5aR1 (Huber‐Lang *et al*, 2002). Complement anaphylatoxins C3a and C5a are pro‐inflammatory and trigger monocyte and macrophage activation (Bohlson *et al*, 2014). C5a receptor signalling on monocyte‐derived macrophages through production of IL‐6 and TNF‐α can enhance cell susceptibility to infection by certain viruses (Kacani *et al*, 2001). Cytokine release through excessive C5aR1 signalling on pro‐inflammatory macrophages and other leucocytes is thought to contribute to the cytokine storm associated with sepsis and MOF. Furthermore, blockade of complement anaphylatoxin C5a in experimental sepsis virtually prevents the appearance of MOF and improves the outcome (Rittirsch *et al*, 2008).

It is a common observation that chronic cardiopulmonary conditions predispose to severe COVID‐19. One theory is that hypoxia in these conditions is a stimulant to complement activation. A rabbit model of ARDS examined the impact of hypoxia (Nuytinck *et al*, 1986). It showed that the combination of hypoxia and activated complement components caused aggregation and degranulation of neutrophils, with consequent lysis and extensive microvascular damage. The characteristic microvascular inflammation and MOF in these animals signified the potent effect of this combination in the pathogenesis of ARDS

## The evidence for complement engagement in COVID‐19

Recent research has shown that SARS‐CoV‐2 S protein is heavily glycosylated with residues that are rich in L‐fucose or D‐mannose (Walls *et al*, 2020; preprint: Watanabe *et al*, 2020) (Fig 2). Hypothetically, the virus could activate the complement pathway through interaction with a lectin, such as CL‐11 or FCN‐1. These are expressed at the alveolar epithelium (Liu *et al*, 2005; Keshi *et al*, 2006; Motomura *et al*, 2008; Hansen *et al*, 2018) and also in the circulation (Honore *et al*, 2008; Bayarri‐Olmos *et al*, 2018). Viral particles entering the circulation would come into contact with MBL as well as the ficolins and CL‐11. It is therefore plausible that interaction of SARS‐CoV‐2 with these lectins triggers the inflammatory and coagulation cascades in the lung and circulation.

What clinical evidence is there to support lectin pathway engagement in COVID‐19? A non‐peer‐reviewed pre‐print by Gao *et al* examined post‐mortem tissue of COVID‐19 patients with ARDS. They reported finding strong immunohistochemical staining for MBL, MASP‐2, C4, C3 and C5b‐9 in the lung (preprint: Gao *et al*, 2020). They suggested that type I and type II alveolar epithelial cells were main tissue targets for complement deposition. Moreover, the presence of MBL and MASP‐2 infers a role for the lectin pathway in this process though it does not establish causality. Serum C5a levels were elevated in their patients with severe lung disease, providing indication of systemic activation of complement or leakage of the activated fragment from the diseased lung. Simultaneously, Magro *et al* reported on a group of five COVID‐19 patients who died with respiratory failure and possibly coagulopathy (Magro *et al*, 2020). Here, the predominant lesion in the lung was microvascular thrombosis associated with MASP‐2, C4 and C5b‐9 deposition with colocalisation for SARS‐CoV‐2 S protein, but with relative sparing of alveolar cells. Three out of five patients had clinical manifestations consistent with a systemic procoagulant state, including elevated d‐dimers and skin purpura, suggesting the possibility of microvascular thrombotic disorder, which could be triggered by lectin pathway components in the presence of SARS‐CoV‐2. These two studies suggest that more than one pattern of lung injury can occur, though at this stage additional lung biopsy data are needed to support the post‐mortem findings.

In addition, there have been recent reports in small groups of children with COVID‐19 with aggressive multiorgan disease and laboratory evidence of hyper‐inflammatory disease and thrombosis (Riphagen *et al*, 2020; Verdoni *et al*, 2020). Vasculitic lesions and hypercoagulability in these children are strongly suggestive of complement involvement through co‐triggering of the complement and coagulation cascades.

**Figure 4:**
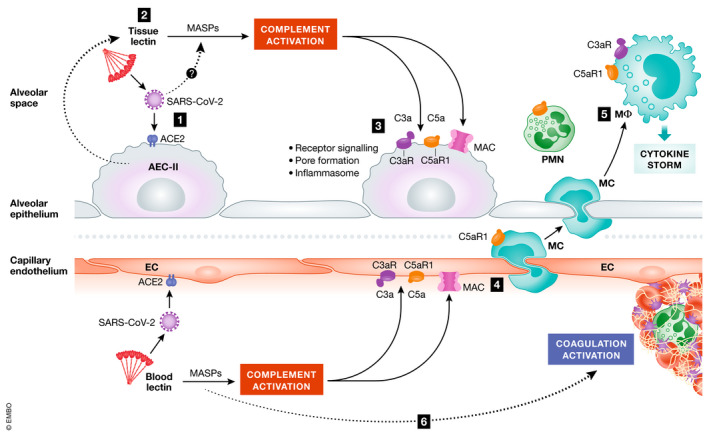
Hypothetical pathway for complement‐mediated inflammation of the pulmonary alveolus in COVID‐19 (1) SARS‐CoV‐2 attaches to type II alveolar epithelial cell (AEC‐II) receptor angiotensin‐converting enzyme 2 (ACE2). (2) Complement activation is initiated upon recognition of viral glycans by lectins (e.g. collectin‐11 and ficolin‐1, which are secreted by AEC‐II) complexed with MBL‐associated serine proteases (MASPs) including MASP‐2. Direct binding of MASP‐2 to the N protein of SARS‐CoV‐2 has also been suggested to initiate lectin pathway activation (preprint: Gao *et al*, 2020). (3) Complement deposition and MAC formation on AECs cause inflammasome activation and cell damage. (4) Release of complement C5a increases vascular permeability and recruitment/activation of polymorphs (PMN) and monocytes (MC) to the alveolus. (5) Monocytes differentiated into inflammatory macrophages (MΦ) overproduce pro‐inflammatory cytokines in response to C3a and C5a stimulation. (6) Endothelial cell (EC) activation by C5a and MAC predisposes to thrombus formation, further enhanced through MBL recognition of viral particles in the vascular compartment leading to cleavage of thrombin and fibrinogen by MASPs.

It is tempting to suggest that alveolar epithelium is the primary site of complement activation following exposure to SARS‐CoV‐2 (Fig 4). Type II alveolar cells can secrete multiple complement factors, including the pattern recognition molecules FCN‐1 and probably CL‐11, as well as being the main cell type targeted by the virus. Complement activation in the presence of these ligand‐recognition molecules and other essential proteins of the lectin complement pathway would drive the formation of C3a, C3b, C5a and C5b‐9, which are the main pro‐inflammatory effectors of the complement cascade. Secondary activation of macrophages that populate the subepithelial space, through C5aR signalling, could enhance the release of cytokines into the local environment and through to the circulating compartment. Since macrophages also express the receptor (ACE2) for SARS‐CoV‐2 and produce complement components, viral invasion of macrophages could potentially amplify the effect of C5a on cytokine release. On the microvascular injury, we would propose that viral particles entering the circulation could trigger complement activation by the MBL pathway. The procoagulant effects of MASP‐1 and MASP‐2 (described earlier) coupled with the prothrombotic effects of C5a and MAC on endothelium could then predispose to microvascular thrombosis. Further studies are needed to test this perception.

A novel investigation on the transcriptional profile of SARS‐CoV‐2‐infected human alveolar epithelial cells identified a curious phenomenon in a pre‐print (Blanco‐Melo *et al*, 2020). Compared to other respiratory viruses, SARS‐CoV‐2 elicited a muted inflammatory response that included the type I and type III interferons and numerous chemokines and cytokines (Blanco‐Melo *et al*, 2020). In complete contrast, complement gene expression was markedly increased in the SARS‐CoV‐2‐infected cells (Blanco‐Melo *et al*, 2020). Either this could mean that epithelial production of complement is an effective way of clearing the virus, or it could imply the organism is complement‐resistant. A number of microbes have acquired characteristics that enable complement evasion or subversion. For example, resistant pathogenic *E*. coli can use complement receptor CD46 to mediate epithelial cell entry (Springall *et al*, 2001; Li *et al*, 2006). CD46 can function as a receptor for other bacteria and viruses, including measles virus (Schnorr *et al*, 1995). Epstein–Barr virus successfully integrates into lymphoid cells using the complement receptor 2 (CD21) (Smith *et al*, 2020). In a similar way, the role of the complement system in infection by SARS‐CoV‐2 may be counterintuitive since the virus could have mechanisms to resist and/or exploit complement to facilitate cell entry.

Distant effects of COVID‐19 lung disease on other organs could be explained by blood‐borne infection or by the effect of toxic levels of circulating inflammatory mediators. Disseminated intravascular coagulation may also play a role. For example, acute kidney injury (AKI) can occur, which is reported in up to 36.6% of hospitalised patients with COVID‐19 (Hirsch *et al*, 2020). The characteristic lesion includes renal tubule damage with complement deposition, alongside evidence of viral particles in glomerular podocytes and renal tubule cells, as reported in an early pre‐print (preprint: Diao *et al*, 2020). Renal tubule cells, like cardiac and lung cells, express ACE2, which could explain how the viral particles are retained and why the focus of the inflammatory response is on the proximal tubule segments. The proximal tubule epithelial cell is also a prominent local source of CL‐11 and other complement components known to participate in AKI (Farrar *et al*, 2016). Angiotensin‐converting enzyme, another product of the proximal tubule epithelium, can directly cleave C3 to precipitate complement activation by another route (Semis *et al*, 2019). Complement control could therefore have a protective effect on some or all of these processes.

Earlier research on the SARS‐CoV‐1 and MERS‐CoV viruses has provided additional insight into the complement pathways triggered by pathogenic human coronaviruses. Following the SARS‐CoV‐1 outbreak, a number of research groups looked at MBL as a mediator of pathology, with conflicting results (Ip *et al*, 2005; Yuan *et al*, 2005). The low expression variant of MBL was reported as a susceptibility factor for SARS‐CoV‐1 infection (Ip *et al*, 2005; Zhang *et al*, 2005; Tu *et al*, 2015). Among these, the study of Tu *et al* included 932 patients with SARS, which accounted for 12% of the SARS worldwide (Tu *et al*, 2015). A study on SARS‐CoV‐1 demonstrated that the SARS S glycoprotein interacts with MBL at a single asparagine‐linked glycosylation site (Zhou *et al*, 2010), while deposition of complement C4 on SARS‐CoV‐1 was enhanced by MBL (Ip *et al*, 2005). On the contrary, it was demonstrated by other studies that the S glycoprotein did not bind to MBL (Leth‐Larsen *et al*, 2007). In another animal model, namely chicken coronavirus infectious bronchitis virus (IBV), the antiviral activity of chicken MBL was exhibited through its binding to the spike S1 glycoprotein of the virus by its CRD in a Ca^2+^‐dependent manner (Zhang *et al*, 2017).

The comparative study by Gao and colleagues examined the N proteins of SARS‐CoV‐1, MERS‐CoV and SARS‐CoV‐2 for ability to activate the lectin pathway (preprint: Gao *et al*, 2020). Their pre‐print reported direct binding of the N proteins to MASP‐2, the key serine protease of the lectin pathway. MASP‐2 cleaves the complement components C4 and C2 to generate C3 convertase (Farrar *et al*, 2016); MASP‐2 can also directly cleave C3 (Schwaeble *et al*, 2011). They demonstrated that this enzymatic activity of MASP‐2 was enhanced in the presence of N protein (preprint: Gao *et al*, 2020). These findings, if confirmed, would suggest that pattern recognition of viral glycoproteins is important for inducing over‐activity of the downstream inflammatory response mediated by the lectin pathway, and also highlight MASP‐2 as a potential therapeutic target that is physically associated with all of the major collectins.

Current mouse models offer limited value for investigating SARS‐CoV‐2 infection. This is because murine ACE2, the principal receptor for the virus, does not have a high degree of homology with the human ACE2 which binds successfully to SARS‐CoV‐2 (Wang *et al*, 2020a). Thus, infectivity studies using HeLa cells that expressed or not ACE2 proteins from humans, Chinese horseshoe bats, civets, pigs and mice showed that SARS‐CoV‐2 is able to use all but the murine ACE2 proteins (Zhou *et al*, 2020). Nonetheless, research with SARS‐CoV‐1, which binds to a higher extent the murine ACE2, has shown that nasally infected mice develop complement activation in the lung, whereas complement C3‐deficient mice were protected from virus‐induced lung injury (Gralinski *et al*, 2018). The protected mice had fewer neutrophils and inflammatory monocytes in their lungs, resulting in lower cytokine and chemokine levels in the lungs and sera (Gralinski *et al*, 2018). Furthermore, a study on ferrets showed that primary infection with SARS‐CoV‐1 leads to upregulation of complement genes including the lectin pathway components MASP1 and ficolin‐1 (Cameron *et al*, 2012); this is in agreement with the study by Blanco‐Melo *et al* with SARS‐CoV‐2 infection.

A murine study on MERS‐CoV emphasised that excessive complement activation may contribute to acute lung injury after infection, while blockade of the complement C5a‐C5a receptor axis alleviated the lung damage (Jiang *et al*, 2018). Anti‐C5aR1 antibody treatment in infected mice even led to decreased pulmonary viral replication (Jiang *et al*, 2018). These findings were reflected in an early report of an ongoing clinical study, where two COVID‐19 patients with ARDS began to improve only after treatment with recombinant anti‐C5a antibody (preprint: Gao *et al*, 2020).

## Therapeutic opportunities

With the accumulation of data supporting an excessive inflammatory response, in part due to over activation of the complement system, attention has turned to the potential use of therapeutic complement inhibitors that are on the market and in various stages of development (Table 1). For a comprehensive list of complement therapies, see publication by Zelek *et al* (2019). Among these are antibodies, proteins, recombinant proteins, peptides, small molecules and siRNA that target specific components of the complement pathway or complement activation per se. As a more detailed understanding of the host/pathogen interface and disease immunopathology is acquired, this will inform treatment options such as whether therapy should be by local or systemic administration or selective for a specific complement component (such as C5a), complement receptor, or whether complete inhibition of the entire complement system should be targeted (targeting C3).

**Table 1:** Examples of therapeutic complement inhibitors and stage of development

Name of Drug (Company/Trial)	Mechanism of action	Indication	Stage of development	Mode of Administration	Reference
Berinert (CSL Behring UK Ltd) Cinryze (Shire Pharmaceuticals Ltd)	C1 esterase Inhibitor (C1‐INH) from human plasma, inactivates C1s and C1r	‐Hereditary angioedema (HAE)	On the market	Intravenous	
Pegcetacoplan/APL‐2 (Apellis Pharmaceuticals Inc.)	C3 inhibitor, pegylated derivative of Compstatin. Inhibits C3 cleavage	‐Paroxysmal nocturnal haemoglobinuria (PNH) ‐Geographic atrophy (GA) secondary to age‐related macular degeneration (AMD)	FDA fast track designation	Subcutaneous self‐administration Intravitreal	NCT03500549 NCT03525613
AMY‐101 (Amyndas Pharmaceuticals)	Peptide inhibitor binds C3 to prevent cleavage to C3a/C3b	‐Chronic periodontal inflammation—gingivitis	Phase IIa	Subcutaneous	NCT03694444
Mirococept (Adprotech/EMPIRIKAL Trial)	Membrane‐inserting recombinant human CR1 Inhibits C3 and C5 convertases to modify local pro‐inflammatory/procoagulant environment.	‐Ischaemia reperfusion injury (IRI) in kidney transplantation.	Phase IIb	*Ex vivo* perfusion via renal artery	Kassimatis *et al* (2017)
Eculizumab or Soliris (Alexion Pharmaceuticals Inc.) Ultomiris (ravulizumab, long‐acting, Alexion Pharmaceuticals) SOLID‐C19 Trial	Anti‐C5 antibody blocks cleavage to C5a/C5b. Inhibits C5b‐9 assembly	‐PNH ‐Atypical haemolytic uraemic syndrome (aHUS) ‐ COVID‐19	On the market Expanded access	Intravenous	NCT04288713
Zilucoplan (Ra Pharmaceuticals)	C5 binding peptide, blocks C5a/C5b production and C5b‐9 assembly	‐Generalised myasthenia gravis (gMG),	Phase III	Subcutaneous self‐administration	NCT04115293
Cemdisiran or ALN‐CC5 (Alnylam Pharmaceuticals)	siRNA silences hepatocyte‐expressed C5	‐IgA nephropathy	Phase II	Subcutaneous	NCT03841448
CCX168 or Avacopan (ChemoCentryx)	Anti‐C5aR antagonist. Small molecule.	‐ANCA‐associated renal vasculitis (AARV)	Phase III	Oral	NCT02994927
IFX‐1 (InflaRX, Europe) BDB‐001 (China) (Staidson Biopharmaceuticals Co., Ltd)	Anti‐C5a antibody inhibits activity of C5a	‐ Severe COVID‐19 ‐ Mild COVID‐19^a^ ‐ Severe and critical COVID‐19^b^	Phase II/III Phase II	Intravenous Intravenous	NCT04333420 (preprint Gao *et al*, 2020) 2020L00003
OMS721 or Narsoplimab (Omeros)	Anti‐MASP‐2 antibody, targets the lectin pathway	‐aHUS Haematopoietic stem cell transplant‐associated thrombotic microangiopathy (HSCT‐TMA) ‐IgA nephropathy	Phase IIIPhase IIIPhase III	Intravenous or subcutaneous	NCT03205995NCT02222545NCT03608033

a Multicentre, randomised double‐blind placebo‐controlled trial.

b Open‐label two‐cohort clinical trial. The first two cases reported showed alleviated pneumonia 20 and 12 days after the first dose.

A trial of C5‐specific antibody eculizumab for severe COVID‐19 has begun (SOLID‐C19 NCT04288713). This is supported by preliminary data obtained using eculizumab as an off‐label treatment for four patients with severe COVID‐19 in combination with anti‐coagulant therapy, antiviral therapy, hydroxychloroquine, an antibiotic, vitamin C and non‐invasive ventilation. All patients recovered, and mean duration of the disease was 12.8 days (Diurno *et al*, 2020).

In addition, trials using a more targeted approach have been instigated using antibody blockade of the C5a fragment, while leaving the terminal effector (C5b‐9) intact, which may be beneficial (BDB‐001, China 2020L00003 (preprint: Gao *et al*, 2020), IFX‐1 Europe NCT04333420).

Indications that the coronavirus N protein binds MASP‐2 and the detection of MASP‐2 staining in post‐mortem lung sections from COVID‐19 patients (Magro *et al*, 2020; preprint: Gao *et al*, 2020) may support a trial of therapeutic anti‐MASP‐2 antibody (such as narsoplimab (OMS721)) to suppress lectin pathway activation.

The sheer severity of the inflammatory response and cytokine storm justifies the use of therapeutic targeting of the meeting point of all three activation pathways, i.e. C3. This could potentially be achieved using a derivative of Compstatin, a cyclic peptide that binds C3 and prevents the action of C3 convertases (Mastaglio *et al*, 2020). Another option is the recombinant protein Mirococept (Smith & Smith, 2001), which is a membrane‐localising complement inhibitor based on a recombinant fragment of human complement receptor 1 (CR1; or CD35), attached to a membrane‐binding peptide tail. The tail consists of a synthetic positively charged peptide that interacts with anionic phospholipids, joined to a membrane‐inserting myristoyl tail (Smith & Smith, 2001; Pratt *et al*, 2003). It retains all biological activity of native CR1 but is approximately a tenth of the size (24 kD) and binds cells to locally block complement activation, by inhibition of C3 and C5 convertases (Masaki *et al*, 1992). In principle, the local delivery of this potent therapeutic complement inhibitor could maximise localisation in the lung where the utmost inflammation occurs. Furthermore, the novel membrane‐inserting tail should enable local binding at high concentration (Smith, 2002) while avoiding unwanted side effects of systemic delivery. It should be noted that Mirococept is transferred in the circulation by erythrocytes also expressing native CR1, whose expression varies by up to 10‐fold among healthy individuals (Herrera *et al*, 1998) and, during the progressive phase of SARS, was reported to drop significantly (Wang *et al*, 2005), possibly due to the release of small vesicles from the erythrocyte membrane leading to its proteolytic cleavage, as has been described previously in other viral infections (Pascual *et al*, 1994). Restored levels of erythrocyte CR1 function in immune complex clearance could be an additional benefit of delivering a recombinant fragment of CR1 (Mirococept) to these patients.

**Pending issues**

(i) Interaction between SARS‐CoV‐2 and named collectins/ficolins (inferred from data with other coronaviruses) and capacity to activate the complement cascade needs to be confirmed.
(ii) The relative importance of locally produced lectins and complement components (namely those secreted by type II alveolar epithelial cells, challenged by SARS‐CoV‐2), as opposed to circulating components, should be investigated, because this may inform the development of effective complement inhibition strategies.
(iii) Clinical trial outcomes with different complement inhibitors will provide proof‐of‐concept data concerning the role of complement in severe COVID‐19 and the site‐/pathway‐/molecule‐specific requirements.

## Conclusions

The causative agent of COVID‐19 has an abundant display of glycoproteins on its outer surface, and these could form potential ligands for several pattern recognition molecules (e.g. collectins) that are produced in the lungs along with other complement proteins, notably by type II alveolar cells and macrophages. The early findings in post‐mortem lung tissue from COVID‐19 patients are consistent with complement deposition triggered by the lectin complement pathway. Treatment with complement inhibitors against C3 or C5 or relevant activating pathways could potentially stem the downstream inflammatory response and capillary leak, assuming adequate tissue penetration of drug to the site of complement activation. This could reduce lung inflammation and secretion volume and deliver increased blood oxygenation and reduced need for respiratory support. It might also reduce the systemic complications of ARDS including MOF and coagulopathy mediated by the lectin pathway. There is urgency to test this hypothesis by clinical trial with phase II‐ or phase III‐tested therapeutic agents.

### Conflict of interest

SS consults for Omeros and Alexion Pharmaceuticals Inc. on therapeutic targets in the complement system.

## For more information

(i) https://complement.org.uk
(ii) https://www.isv-online.org
(iii) https://www.who.int/emergencies/diseases/novel-coronavirus-2019/global-research-on-novel-coronavirus-2019-ncov
